# Structural and Quantitative Characterization of Mucin-Type *O*-Glycans and the Identification of *O*-Glycosylation Sites in Bovine Submaxillary Mucin

**DOI:** 10.3390/biom10040636

**Published:** 2020-04-20

**Authors:** Jihye Kim, Changsoo Ryu, Jongkwan Ha, Junmyoung Lee, Donghwi Kim, Minkyoo Ji, Chi Soo Park, Jaeryong Lee, Dae Kyong Kim, Ha Hyung Kim

**Affiliations:** 1 Biotherapeutics and Glycomics Laboratory, College of Pharmacy, Chung-Ang University, Seoul 06974, Korea; non1172@naver.com (J.K.); lectin@hanmail.net (C.R.); skyview0604@nate.com (J.H.); 123jun123@naver.com (J.L.); kimdh2458@naver.com (D.K.); changlyong2@naver.com (M.J.); ack11111@naver.com (C.S.P.); ioi_1007@naver.com (J.L.); 2 Department of Environmental & Health Chemistry, College of Pharmacy, Chung-Ang University, Seoul 06974, Korea

**Keywords:** bovine submaxillary mucin, mucin-type *O*-glycan, *O*-glycosylation site

## Abstract

Bovine submaxillary mucin (BSM) is a gel-forming glycoprotein polymer, and Ser/Thr-linked glycans (*O*-glycans) are important in regulating BSM’s viscoelasticity and polymerization. However, details of *O*-glycosylation have not been reported. This study investigates the structural and quantitative characteristics of *O*-glycans and identifies *O*-glycosylation sites in BSM using liquid chromatography–tandem mass spectrometry. The *O*-glycans (consisting of di- to octa-saccharides) and their quantities (%) relative to total *O*-glycans (100%; 1.1 pmol per 1 μg of BSM) were identified with 14 major (>1.0%), 12 minor (0.1%–1.0%), and eight trace (<0.1%) *O*-glycans, which were characterized based on their constituents (sialylation (14 *O*-glycans; 81.9%, sum of relative quantities of each glycan), non-sialylation (20; 18.1%), fucosylation (20; 17.5%), and terminal-galactosylation (6; 3.6%)) and six core structure types [Gal-GalNAc, Gal-(GlcNAc)GalNAc, GlcNAc-GalNAc, GlcNAc-(GlcNAc)GalNAc, and GalNAc-GalNAc]. *O*-glycosylation sites were identified using *O*-glycopeptides (bold underlined; _56_**S**GE**T**R**TS**VI, _259_**S**H**SSS**GR**S**R**T**I, _272_G**S**P**SS**V**SS**AEQI, _307_RP**S**YGAL, _625_Q**T**LGPL, _728_**T**M**TT**R**TS**VVV, and _1080_RPEDN**T**AVA) obtained from proteolytic BSM; these sites are in the four domains of BSM. The gel-forming mucins share common domain structures and glycosylation patterns; these results could provide useful information on mucin-type *O*-glycans. This is the first study to characterize *O*-glycans and identify *O*-glycosylation sites in BSM.

## 1. Introduction

Mucins are macromolecular (molecular mass, 0.4–4 MDa) glycoprotein constituents of mucus [1], and are highly modified with Ser/Thr-linked glycans (*O*-glycans) [2]. Mucins play a role in the protection of the host infection [2,3] and are involved in various functions, including gel-formation, viscoelasticity, hydration, and lubrication [3], and *O*-glycosylation is important in the regulation of these properties.

The biosynthesis of mucin-type *O*-glycosylation is initiated by *N*-acetylgalactosamine (GalNAc) attached to the hydroxyl moiety of Ser or Thr, and it is usually extended to form one of several common core structures [4]. These mucin-type *O*-glycans consist of GalNAc, *N*-acetylglucosamine (GlcNAc), fucose (Fuc), galactose (Gal), and sialic acids (SAs), including *N*-acetylneuraminic acid (Neu5Ac) and *N*-glycolylneuraminic acid (Neu5Gc).

*O*-glycans are hydrophilic and negatively charged due to the SA [5], and they are essential for extended conformation; forming rod-like structures; binding pathogens such as bacteria, fungi, virus, and other microbes; preventing protease cleavage; preventing antibodies from recognizing protein epitopes; adhesiveness; polymerization of mucins [3,6]. The majority of mucin research has focused on the effects of *O*-glycans on a specific mucin function [3,5].

Bovine submaxillary mucin (BSM), which is a natural gel-forming mucin from bovine submaxillary glands, is a macromolecular *O*-glycosylated protein (4 MDa; determined by light-scattering) [7]. The physicochemical properties of BSM include high viscosity, biocompatibility, and amphiphilicity [3,5]; therefore, BSM is used in various biomaterial and biomedical applications [3], such as coating pathogenic microbes to reduce their growth [8], stabilization of oil-water emulsions [9], solubilization of hydrophobic drugs to improve bioavailability or membrane penetration, and biological hydrogel formation [10] for sustained-release hydrophobic drug delivery systems [11].

The amino acid (AA) sequence of the BSM protein (1–1589) has five distinct protein domains that are numbered starting from the C-terminus [12]. Each BSM domain has different functions. Domain I (AA 1356–1589) is Cys-rich and affects the conserved β-strand structure as well as dimerization, which helps form the larger protein complex. Domains II (AA 1048–1355), III (AA 344–1047), IV (AA 208–343), and V (AA 1–207) are Ser/Thr-rich and predicted to be potential *O*-glycosylation sites.

Generally, *O*-glycans have smaller and more diverse structures than *N*-glycans; thus, *O*-glycans and their attachment sites are difficult to analyze because there is no universal *O*-glycan-releasing enzyme, *O*-glycan peeling can occur during chemical de-*O*-glycosylation, and there is no consensus amino-acid motif for *O*-glycosylation sites [2].

Recently, *O*-glycan analysis has been performed using ultra-performance liquid chromatography (UPLC) and liquid chromatography (LC)–electrospray ionization (ESI)–high-energy collisional dissociation (HCD)–tandem mass spectrometry (MS/MS) [13] based on a high resolution, a high mass accuracy (<10 ppm), and a milder ionization source for ESI [14]. Additionally, *O*-glycopeptide analysis has been performed using nano-LC–HCD–MS/MS to generate structural information [15] because it is effective at identifying *O*-glycopeptides with informative fragment ions, including distinct Y_1_ (peptide + HexNAc_1_) ions derived from glycosidic bond cleavages.

De-*O*-glycosylated BSM shows no biological function [3]; thus, *O*-glycans are essential for BSM. However, structural characterization and quantification of *O*-glycans of BSM and their attachment sites have not been reported.

In this study, BSM *O*-glycans were identified using non-reductive β-elimination with two different fluorescent labels, and the relative quantities of each glycan and their total concentrations were obtained. *O*-glycans were then characterized based on their constituents and core structure types. *O*-glycosylation sites were identified using enriched glycopeptides obtained from proteolytic BSM. The functional roles of the identified mucin-type *O*-glycans and attachment sites in BSM domains are briefly discussed.

## 2. Materials and Methods

### 2.1. Purity

BSM (Type I-S) was purchased from Sigma-Aldrich (St. Louis, MO, USA). The purity was evaluated with sodium dodecyl sulfate-polyacrylamide gel electrophoresis (SDS-PAGE) using a 12% polyacrylamide gel, as previously described [16]. Molecular mass markers were purchased from Bio-Rad Laboratories (Hercules, CA, USA), and the gel was stained with Coomassie blue R-250.

### 2.2. O-Glycan Preparation

*O*-glycans were released from BSM using a GlycoProfile non-reductive β-Elimination Kit (Sigma-Aldrich). Briefly, the β-elimination reagent mixture was added to BSM (1 mg/mL) and incubated at 4 °C for 18 h, and 0.1 M HCl was added to neutralize the pH. Peptides and salts were removed using a graphitized carbon cartridge (Alltech, Deerfield, IL, USA) [17] that was pre-equilibrated with 80% acetonitrile containing 0.1% trifluoroacetic acid (Sigma-Aldrich) and equilibrated with water. *O*-glycans were eluted with 25% acetonitrile containing 0.05% trifluoroacetic acid and lyophilized.

The released *O*-glycans were derivatized with 2-aminobenzamide (AB; Sigma-Aldrich) [18] or procainamide (ProA; Sigma-Aldrich) [19] by reductive amination. AB (5 mg) and sodium cyanoborohyde (6 mg), and ProA hydrochloride (16.2 mg) and sodium cyanoborohyde (9.7 mg) were each dissolved in dimethyl sulfoxide/acetic acid (7:3, *v*/*v*). Each solution was added separately to each of the lyophilized *O*-glycan samples and incubated at 65 °C for 3 h. Excess fluorophore was removed with microcrystalline cellulose (Sigma-Aldrich) column chromatography. The column was equilibrated and washed with 10 mL n-butyl/acetic acid/ethanol (100:25:25, *v*/*v*/*v*) solution and eluted with 4 mL ethanol/75 mM ammonium bicarbonate (50:100, *v*/*v*). The eluted AB- or ProA-labeled *O*-glycans were lyophilized and stored at −20 °C until used.

### 2.3. O-Glycan Profile

The AB- or ProA-labeled *O*-glycans were profiled on an ACQUITY UPLC H-Class System (Waters, Milford, MA, USA) equipped with a Quaternary Solvent Manager and Sample Manager coupled to an ACQUITY UPLC fluorescence detector (Waters). LC separations were performed on an ACQUITY UPLC BEH-Glycan column (1.7 μm, 2.1 × 150 mm; Waters) at 40 °C. The fluorescence was observed at excitation and emission wavelengths of 330 and 420 nm (for AB), and 310 and 370 nm (for ProA). The flow rate was 200 μL/min. The mobile phase consisted of 50 mM ammonium formate, pH 4.4 (A), and 100% acetonitrile (B), and the column was equilibrated with 75% B before sample injection. The samples were separated over a 25%–50% gradient of A for 50 min before washing the column with 100% A for 9 min and re-equilibrating the column with 75% B at a constant flow rate of 200 μL/min.

### 2.4. Structure and Quantification of O-Glycans

The AB- or ProA-labeled *O*-glycans were subjected to LC on an Ultimate 3000 LC system (Thermo Fisher Scientific, Waltham, MA, USA) equipped with an ACQUITY UPLC BEH-Glycan column (1.7 μm, 2.1 × 150 mm; Waters). A Q Exactive Hybrid Quadrupole Orbitrap mass spectrometer (Thermo Fisher Scientific) was operated in ESI-positive mode. The mobile phase and separation conditions were the same as those used in UPLC. The *O*-glycans were identified by the elution time of the UPLC chromatogram and the presence of the characteristic oxonium ions of *O*-glycans in the MS spectrum. The quantity (%) of each glycan relative to total *O*-glycans (as 100%) was determined from the extracted ion chromatogram (EIC) areas for all observed charge states in LC–ESI–HCD–MS/MS.

### 2.5. Glycopeptide Preparation

BSM was reduced and alkylated with dithiothreitol and iodoacetamide in denaturing buffer (1.5 M Tris-HCl, 10 mM EDTA, and 8 M urea) and digested with proteinase K (Sigma-Aldrich) in 50 mM ammonium bicarbonate (pH 8.0) at 37 °C for 18 h. Glycopeptides from digested BSM were loaded onto a Sep-Pak C18 1cc Vac cartridge (Waters), and the peptides were eluted with 10% isopropanol in 5% acetic acid to selectively enrich glycopeptides.

### 2.6. O-Glycosylation Site Analysis

Enriched glycopeptides from BSM were trapped on a C18 Acclaim PepMap RSLC column (3.0 μm, 75 μm × 20 mm; Thermo Fisher Scientific) using a loading pump solvent composed of 2% acetonitrile and 0.05% formic acid in water. The trapped glycopeptides were separated on an EASY-Spray PepMap C18 column (2.0 μm, 75 μm × 500 mm; Thermo Fisher Scientific) using NC pump solvents for glycopeptide analysis. NC pump solvents consisted of mobile phase A (0.1% formic acid in aqueous solution) and mobile phase B (0.1% formic acid in acetonitrile). Samples were separated with a 2%–40% linear gradient of solvent B over 50 min at a flow rate of 200 nL/min. The mass spectrometry (MS) parameters used were the same as previously reported [19]. The spectral interpretation was carried out according to the procedure of Segu et al. [20]. Briefly, Y_1_ ions of the glycopeptides were indicated by distinct peaks as the most abundant ion in the MS/MS spectrum, and the other peaks were determined as their composition through the different mass values based on Y_1_.

## 3. Results

### 3.1. O-Glycan Profiles

BSM purity was assessed using SDS-PAGE. A protein band corresponding to BSM (confirmed in our previous report [18]) with a purity of >98% (data not shown) was identified. Ultrafiltration was performed to remove any small molecules and salts.

*O*-glycans were released from BSM by non-reductive β-elimination, and the reducing ends were labeled with AB or ProA. Non-reductive β-elimination produces free *O*-glycans with a reducing end and minimizes the peeling reaction in ammonia conditions to release *O*-glycans from glycoproteins [21]. AB and ProA have been used for reductive amination; of these, AB is the most frequently used, and ProA has a stronger fluorescence intensity and better ionization efficiency [22].

**Figure 1:**
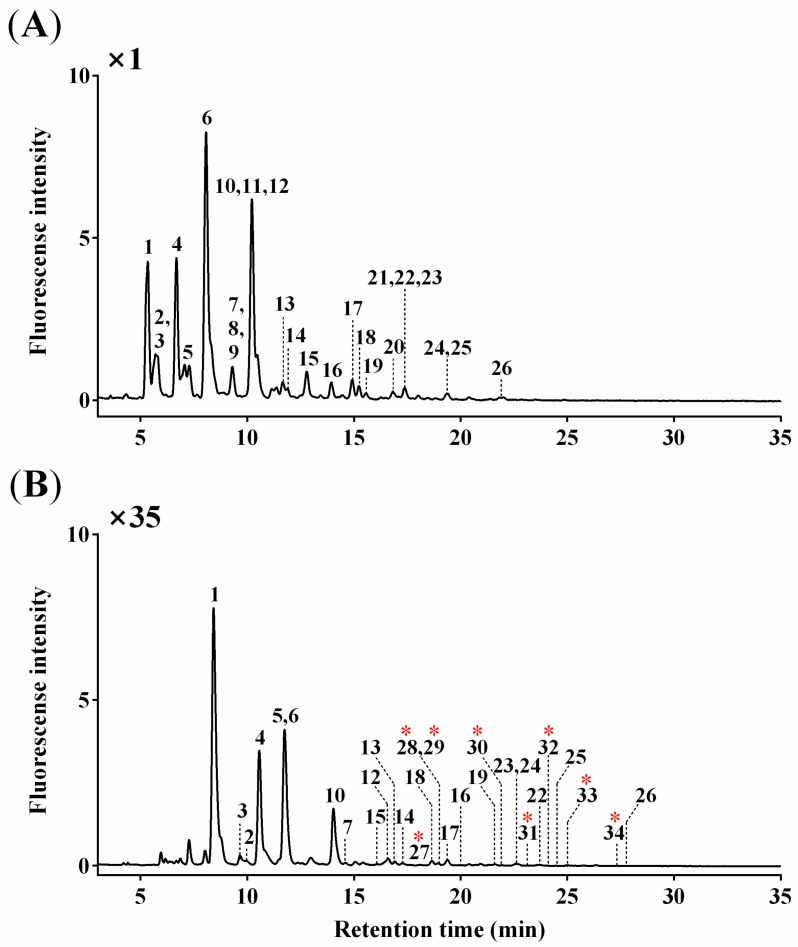
UPLC chromatograms of (**A**) AB-labeled (major and minor) *O*-glycans (Peaks 1–26) and (**B**) ProA-labeled (trace) *O*-glycans (Peaks 27–34; red asterisks) from BSM.

*O*-glycan profiles were obtained from UPLC chromatogram (Figure 1), and a total of 34 *O*-glycan peaks were identified: 26 AB- and 8 ProA-labeled *O*-glycans. The overall chromatographic patterns were similar for both AB- and ProA-labeled *O*-glycans except for a slightly different retention time (~3 min). ProA-labeled *O*-glycans showed an approximately 35-fold higher fluorescence intensity than an equal amount of AB-labeled *O*-glycans.

Six peaks corresponding to 15 *O*-glycans co-eluted with others (Peak 2 with Peak 3, Peak 7 with Peaks 8 and 9, Peak 10 with Peaks 11 and 12, Peak 21 with Peaks 22 and 23, and Peak 24 with Peak 25 for AB-labeled *O*-glycans; Peak 28 with Peak 29 for ProA-labeled *O*-glycans). Structural and quantitative analyses were subsequently conducted using LC–ESI–HCD–MS/MS, as described in the following sections.

### 3.2. O-Glycan Structures

*O*-glycan structures were analyzed with an MS/MS fragmentation pattern generated by LC–ESI–HCD–MS/MS. The MS/MS spectra of *O*-glycans, which are oxonium ions with a mass error of <5.0 ppm, were clearly observed in all fragmentation patterns.

**Figure 2:**
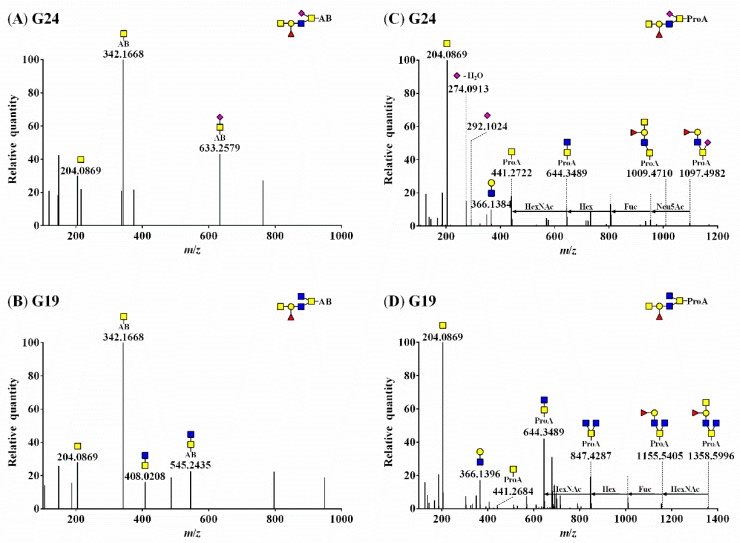
HCD–MS/MS spectra of AB-labeled *O*-glycans (**A**) G24 (theoretical *m*/*z* = 1347.5309) and (**B**) G19 (theoretical *m*/*z* = 1259.5148) and ProA-labeled *O*-glycans (**C**) G24 (theoretical *m*/*z* = 1446.6357) and (**D**) G19 (theoretical *m*/*z* = 1358.6196). ■, GlcNAc; ■, GalNAc; ●, Gal; ▲, Fuc; ◆, Neu5Ac; ◇, Neu5Gc.

Figure 2 shows the MS/MS fragmentation of *O*-glycans labeled with AB (A and B) and ProA (C and D), indicated by G24 (Neu5Ac_1_Fuc_1_Gal_1_GlcNAc_1_GalNAc_2_) and G19 (Fuc_1_Gal_1_GlcNAc_2_GalNAc_2_). The MS/MS spectra revealed the presence of [*N*-acetylhexosamine(HexNAc)-AB]^+^ (theoretical *m*/*z* = 342.1660; observed *m*/*z* = 342.1668 in Figure 2A,B) or [HexNAc-ProA]^+^ (theoretical *m*/*z* = 441.2707; observed *m*/*z* = 441.2722 in Figure 2C and *m*/*z* = 441.2684 in Figure 2D) oxonium ions.

The informative fragmentation ions could not be clearly discerned in AB-labeled *O*-glycans, rendering their structure difficult to elucidate (Figure 2A,B). However, oxonium ions and fragment ions, including Fuc and SA, were identified (Figure 2C,D), and the precursor ions of *O*-glycan were confirmed for ProA-labeled *O*-glycans (Figure 2D and Figure S1). The oxonium ions derived from Neu5Ac were detected at *m*/*z* 292.1024 ([Neu5Ac]^+^) and *m*/*z* 274.0913 ([Neu5Ac−H_2_O]^+^). Two fragment ions containing Fuc (observed *m*/*z* = 1009.4710) and both Fuc and Neu5Ac (observed *m*/*z* = 1097.4982) were detected (Figure 2C). The MS/MS spectrum of [Fuc_1_hexose(Hex)_1_HexNAc_4_-ProA]^+^ revealed a fragment ion containing Fuc (observed *m*/*z* = 1155.5405) and the fragment ion of the precursor ion itself (observed *m*/*z* = 1358.5996) (Figure 2D).

These results indicate that the EIC of ProA-labeled *O*-glycans were more efficiently ionized than those of AB-labeled *O*-glycans, and this can be explained by the high proton affinity of the ProA basic tail [2-(diethylamino)ethyl group], which contributes to ESI–MS/MS sensitivity in positive mode [23]. All 34 *O*-glycan structures and corresponding peak numbers in the UPLC chromatogram and their mass data are summarized in Table 1.

### 3.3. Quantitative Characterization of O-Glycans

The quantity (%) of each glycan relative to total *O*-glycans (as 100%) was determined from the EIC areas generated by LC–ESI–HCD–MS/MS (Table 1). All *O*-glycans showed a high mass accuracy of <10.0 ppm. The singly charged precursor ions were obtained from AB-labeled *O*-glycans, and ProA-labeled *O*-glycans produced singly or doubly charged precursor ions, enabling the detection of trace *O*-glycans.

The 34 *O*-glycans were characterized according to their relative quantities: 14 major (relative quantity >1.0% for each) and 12 minor (0.1%–1.0%) *O*-glycans obtained from AB-labeling and eight trace (<0.1%) *O*-glycans obtained from ProA-labeling (Figure 1). The AB-labeled *O*-glycans consisted of di- (2 *O*-glycans; 20.3%, sum of relative quantities of each glycan), tri- (5; 56.4%), tetra- (7; 11.4%), penta- (4; 7.9%), hexa- (5; 3.4%), hepta- (2; 0.5%), and octa- (1; 0.1%) saccharides. The ProA-labeled *O*-glycans consisted of hexa- (3; <0.1%), hepta- (4; <0.1%), and octa- (1; <0.1%) saccharides (Table 1).

The 34 *O*-glycans were also characterized according to their SA-containing constituents as follows: sialylated (14; 81.9%) including *O*-glycans containing Neu5Ac (8; 49.0%) and Neu5Gc (6; 32.9%) and non-sialylated (neutral) *O*-glycans (20; 18.1%). Additionally, the 34 *O*-glycans were characterized according to their Fuc-containing constituents or terminal-Gal as follows: fucosylated *O*-glycans (20; 17.5%) comprising mono- (11; 14.3%), di- (8; 3.1%), and tri- (1; 0.1%) *O*-glycans, and terminal-galactosylated *O*-glycans (6; 3.6%) (Table 1).

The 34 *O*-glycans were characterized according to their core structures [5,24] as follows: Core 1 Gal-GalNAc (3; 3.6%), Core 2 GlcNAc-(Gal)-GalNAc (12; 14.1%), Core 3 GlcNAc-GalNAc (9; 36.3%), Core 4 GlcNAc-(GlcNAc)-GalNAc (7; 5.2%), and Core 5 GalNAc-GalNAc (1; 20.5%) with the exception of 2 disaccharides, G1 (9.3%) and G4 (11.0%) (Table 1).

Fluorescent labeling can directly quantify *O*-glycans based on their intensities due to the stoichiometric attachment of one fluorescent label per *O*-glycan [25]. The fluorescence intensity of the most distinct *O*-glycan peak, G6, was selected from the UPLC chromatogram (Figure 1A) due to the multiple glycans that can exist in one peak, and calculated as 0.35 pmol using a linear calibration curve (r^2^ = 0.99) generated from AB concentrations (0.09–1.56 pmol) (data not shown). The others were calculated using their relative quantities (Table 1) from the concentration (0.35 pmol) of G6 (32.6%) because all glycans can be extracted as a single peak when the LC–ESI–HCD–MS/MS is used. The total concentration of all *O*-glycans was 1.1 pmol per 1 μg BSM.

### 3.4. Glycopeptides Enrichment

Enriched glycopeptides were prepared in a Sep-Pak C18 1cc Vac cartridge containing hydrophobic, reverse-phase, and silica-based bonded phases using four elution conditions: (1) 10% isopropanol, (2) 20% isopropanol, (3) 40% isopropanol, and (4) 95% isopropanol, each in 5% acetic acid.

Total ion chromatograms (TICs) produced in LC–ESI–HCD–MS/MS was similar in all conditions; however, the largest amounts of total ions in the MS spectra were obtained by eluting with 10% isopropanol in 5% acetic acid (data not shown). The EIC ionic value differed distinctly depending on the condition: (1) 2.21 × 10^7^ (10% isopropanol in 5% acetic acid), (2) 3.06 × 10^4^ (20% isopropanol in 5% acetic acid), (3) 2.83 × 10^4^ (40% isopropanol in 5% acetic acid), and (4) 1.84 × 10^4^ (95% isopropanol in 5% acetic acid). Consequently, Elution Condition (1)—10% isopropanol in 5% acetic acid—indicated a 1000-fold higher ion value when compared to other conditions, and this condition was applied to enrich the glycopeptides.

### 3.5. O-Glycopeptide Structures

To identify sites at which *O*-glycans attach to BSM, glycopeptides obtained from proteinase K digestion of reduced and alkylated BSM were enriched and analyzed by nano-LC–HCD–MS/MS. The 1589 AA sequence of BSM was based on the previous report [12].

**Figure 3:**
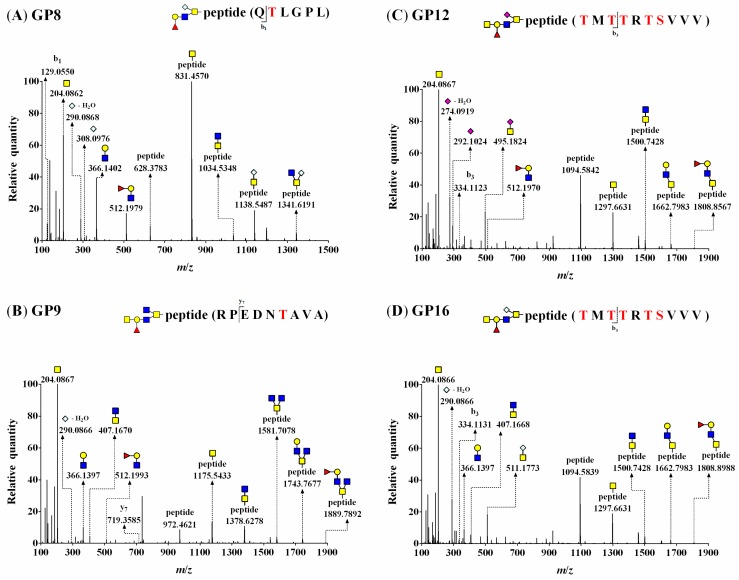
HCD–MS/MS spectra of (**A**) GP8 (G21 attached to QTLGPL, theoretical *m*/*z* = 825.3668), (**B**) GP9 (G19 attached to RPEDNTAVA, theoretical *m*/*z* = 1046.9550), (**C**) GP12 (G24 attached to TMTTRTSVVV, theoretical *m*/*z* = 1152.0195), and (**D**) GP16 (G33 attached to TMTTRTSVVV, theoretical *m*/*z* = 1160.0169) *O*-glycopeptides. All glycopeptide masses were observed as doubly charged forms, and the precursor ions of *O*-glycopeptides are shown in the MS spectra with full scan mode in Figure S2. A red S or T indicates an *O*-glycosylation site of BSM. ■, GlcNAc; ■, GalNAc; ●, Gal; ▲, Fuc; ◆, Neu5Ac; ◇, Neu5Gc.

As shown in Figure 3, the MS/MS spectra showed common oxonium ions and fragment ions, indicating the presence of *O*-glycan constituents: [HexNAc]^+^, [Neu5Gc–H_2_O]^+^, [Neu5Gc]^+^, and [Fuc_1_Hex_1_HexNAc_1_]^+^ (theoretical *m*/*z* = 204.0867 (Figure 3A–D), *m*/*z* = 290.0870 (Figure 3A,B,D), *m*/*z* = 308.0976 (Figure 3A), *m*/*z* = 512.1974 (Figure 3A–C)) with a mass error of <5.0 ppm. The glycosylation sites included a peptide (Y_0_) ion and a peptide with a HexNAc (Y_1_) ion that was observed as the predominant peak at *m*/*z* 831.4570 (Figure 3A) or *m*/*z* 1175.5433 (Figure 3B) in the MS/MS spectra. Additionally, the spectra provided part of a peptide backbone sequence, yielding masses for b_1_ (observed *m*/*z* = 129.0550) (Figure 3A) and y_7_ (observed *m*/*z* = 719.3585) (Figure 3B).

The MS/MS fragment ions of [peptide + HexNAc_2_]^+^ were observed at *m*/*z* = 1034.5348 (Figure 3A) and 1378.6278 (Figure 3B). These MS/MS spectra subsequently represented differences of the fragmentation pattern, [peptide + HexNAc_1or2_Neu5Gc_1_]^+^ ions at *m*/*z* 1138.5487 and 1341.6191 (Figure 3A), and [peptide + Fuc_0or1_Hex_0or1_HexNAc_3_]^+^ ions at *m*/*z* 1581.7078, 1743.7677, and 1889.7892 (Figure 3B). Thus, these MS/MS spectra of glycopeptides (GP8 and GP9) showed G21 (Neu5Gc_1_Fuc_1_Gal_1_GlcNAc_1_GalNAc_1_) attached to Thr 626 in _625_QTLGPL and G19 (Fuc_1_Gal_1_GlcNAc_2_GalNAc_2_) attached to Thr 1085 in _1080_RPEDNTAVA (Figure 3A,B).

Two *O*-glycopeptides consisting of the same peptide attached to different *O*-glycans are shown in Figure 3C,D. Each MS/MS spectrum shows characteristic oxonium ions derived from SA: [Neu5Ac−H_2_O]^+^ ion and [Neu5Ac]^+^ ion at *m/z* 274.0919 and 292.1024, respectively (Figure 3C), and [Neu5Gc−H_2_O]^+^ ion at *m*/*z* 290.0866 (Figure 3D). Figure 3C,D show that the peak of the Y_0_ ion (observed *m*/*z* = 1094.5842 and 1094.5839) was higher than that of the Y_1_ ion (observed *m*/*z* = 1297.6631). The peptide cleavage sites were detected as b_3_ and identical to those in Figure 3C (observed *m*/*z* = 334.1123) and 3D (observed *m*/*z* = 334.1131).

The *O*-glycan compositions attached to the same peptide were G24 (Figure 3C) and G33 (Figure 3D), and the fragmentation patterns of glycopeptides were almost the same, except for oxonium ions. Additionally, the mass values of the same fragmentation ions [peptide + HexNAc_2_]^+^ and [peptide + Hex_1_HexNAc_2_]^+^ were equal (observed *m*/*z* = 1500.7428 and 1662.7983, respectively; Figure 3C,D). Thus, these MS/MS spectra (GP12 and GP16) showed two *O*-glycans, G24 (Neu5Ac_1_Fuc_1_Gal_1_GlcNAc_1_GalNAc_2_) and G33 (Neu5Gc_1_Fuc_1_Gal_1_GlcNAc_1_GalNAc_2_), attached to _728_TMTTRTSVVV as GP12 and GP16, respectively. All detected glycopeptides were analyzed, and 17 *O*-glycopeptides (a combination of seven glycopeptides attached to 10 kinds of *O*-glycans) of BSM were identified (Table 2).

### 3.6. O-Glycosylation Sites at Four BSM Domains

Nine types of *O*-glycopeptides (GP1–GP9) consisting of three peptide backbones and five *O*-glycans were identified, and three peptide backbones (_307_RP**S**YGAL, _625_Q**T**LGPL, and _1080_RPEDN**T**AVA) showed three *O*-glycosylation sites (bold underlined **S** or **T**), at Ser 309, Thr 626, and Thr 1085, respectively. Additionally, eight types of glycopeptides (GP10–GP17) consisting of four peptide backbones and eight *O*-glycans were also identified, and four peptide backbones (_56_**S**GE**T**R**TS**VI, _272_G**S**P**SS**V**SS**AEQI, _728_**T**M**TT**R**TS**VVV, and _259_**S**H**SSS**GR**S**R**T**I) showed four *O*-glycosylation sites (bold underlined **S** or **T**).

**Figure 4:**
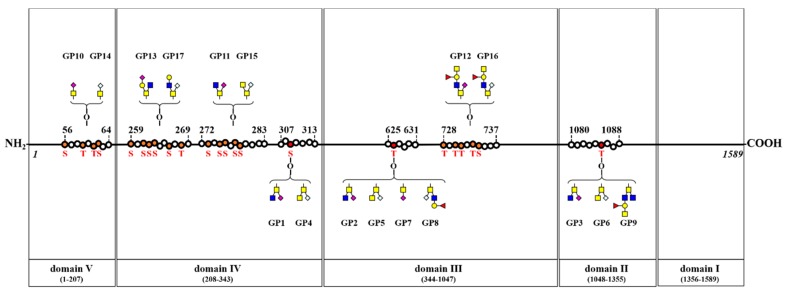
Schematic representation of *O*-glycosylation sites on four domains of BSM. Three *O*-glycosylation sites in each glycopeptide (_307_RPSYGAL, _625_QTLGPL, and _1080_RPEDNTAVA) and four *O*-glycosylation sites in each glycopeptide (_56_SGETRTSVI, _259_SHSSSGRSRTI, _272_GSPSSVSSAEQI, and _728_TMTTRTSVVV). A red S or T indicates an *O*-glycosylation site of BSM. ■, GlcNAc; ■, GalNAc; ●, Gal; ▲, Fuc; ◆, Neu5Ac; ◇, Neu5Gc.

A summary of the glycosylation sites identified in this study is shown in Figure 4; it is based on the previously reported five domains of BSM [12].

Sialylated *O*-glycans are attached at Domains II (1 site), III (2 sites), IV (3 sites), and V (1 site). Fucosylated *O*-glycans are attached at Domains II (1 site) and III (2 sites). A terminal-galactosylated *O*-glycan is attached at Domain IV (1 site). Additionally, G33 was detected in trace amounts of BSM, but GP16 (containing G33) was identified at Domain III.

*O*-glycans containing a core structure of GlcNAc-GalNAc are attached at Domains II (1 site), III (2 sites), and IV (3 sites). One *O*-glycan with a core structure of GlcNAc-(GlcNAc)-GalNAc is attached at Domain II (1 site), and *O*-glycans with a core structure of GalNAc-GalNAc are attached at Domains II (1 site), III (1 site), and IV (2 sites).

## 4. Discussion

*O*-glycans in glycoproteins can alter the structure [26], function [4], and physicochemical properties of the protein to which they are attached [5], and BSM *O*-glycans have been characterized using various analytical methods. Permethylated BSM *O*-glycans have been identified by matrix-assisted laser desorption/ionization (MALDI)–time-of-flight (TOF) MS [27], which yielded different numbers of methylated groups [28], even though MALDI–TOF MS is not appropriate for analyzing *O*-glycans containing SA because these residues tend to be lost during ionization [29]. 1-phenyl-3-methyl-5-pyrazolone (PMP)-labeled *O*-glycans of BSM have been analyzed by LC–MS [30]; however, the separation of isomers and the sensitivity may be adversely affected when two PMP moieties are attached to one *O*-glycan [25], and detection based on ultraviolet absorption is less sensitive than fluorescence detection [21]. Additionally, the average quantities of *O*-glycans of BSM have been identified through repeated experiments using MALDI–TOF MS [27].

The present study identified *O*-glycans, including trace *O*-glycans, of BSM using non-reductive β-elimination with AB and ProA for glycan derivatization and determined their relative quantities using LC–ESI–HCD–MS/MS. AB-labeling is more suitable for glycan quantification than ProA-labeling [31]; thus, the relative quantities of each glycan in the present study were calculated using LC–ESI–HCD–MS/MS data from AB-labeled *O*-glycans. However, glycans in small amounts or trace glycans could be biologically important for many glycoproteins [32]. Therefore, both AB-labeling (for the quantification of *O*-glycans) and ProA-labeling (for the qualification of *O*-glycans) analyses were complementarily used in this study.

Mucin-type *O*-glycans contain mono- to more than 20-saccharides [33], but the present study shows that all BSM *O*-glycans consist of di- to octa-saccharides. Almost all of the *O*-glycans were sialylated, with *O*-glycans containing Neu5Ac and Neu5Gc. Sialylation occurred on di- to hexa-saccharides (not hepta- and octa-saccharides). Additionally, mono- and di- fucosylation occurred on tetra- to hexa-saccharides and hexa- to octa-saccharides, respectively, and terminal-galactosylation occurred on tetra- to hexa-saccharides.

The detailed functional role of each *O*-glycan in mucin is unknown [24], but sialylated, fucosylated, and terminal-galactosylated *O*-glycans play various roles in the functions of mucin or other glycoproteins. For example, sialylated *O*-glycans provide an overall negative charge to the molecule, stabilize protein conformation, increase protein thermal stability, act as a protective barrier, alter protein solubility, entrap pathogens, and enhance the viscosity of mucins [34]. Fucosylated *O*-glycans mediate ligand adhesion, pathogen–host interactions, and cellular processes via signaling mechanisms [35], and terminal-galactosylated *O*-glycans may provide recognition epitopes for galactose-specific lectins [36].

Core 1 Gal-GalNAc and Core 2 GlcNAc-(Gal)-GalNAc structures were found in BSM, which are similar to most human mucins [34], but Core 3 GlcNAc-GalNAc, Core 4 GlcNAc-(GlcNAc)-GalNAc, and Core 5 GalNAc-GalNAc structures, which are less common in other mucins, were also found in BSM. This suggests that BSM has an additional protective function that other mucins may not because these core structures are important in forming mucin barriers that influence host–environment interactions and disease pathogenesis [37].

The present study also identifies *O*-glycosylation sites from *O*-glycopeptides using nano-LC–HCD–MS/MS. Sialylated *O*-glycans, Core 3 GlcNAc-GalNAc, and Core 5 GalNAc-GalNAc structures are extensively attached at BSM, but fucosylated and terminal-galactosylated *O*-glycans are partially attached.

Unfortunately, dense *O*-glycosylations of BSM are resistant to proteolytic digestion, resulting in limited hydrolysis, and the present results reveal one *O*-glycosylated Ser/Thr in each glycopeptide. Thus, further studies are necessary to analyze glycopeptides containing more than two *O*-glycosylated Ser/Thr in each glycopeptide. Additionally, LC–MS/MS with electron-transfer dissociation, which can be used to fragment the peptide backbone and identify AA sequences, is also useful for glycopeptide analysis when combined with the present HCD.

## 5. Conclusions

The gel-forming mucins, including BSM, share common properties, such as domain structures, glycosylation patterns, and biosynthetic pathways [38], and the present results provide useful information regarding the properties, multifunction, and expansion of the range of their applications. This is the first study to characterize and quantify mucin-type *O*-glycans and identify *O*-glycosylation sites within BSM.

## Figures and Tables

**Table 1:** Summary of the structure, relative quantity (%), and mass data of 34 *O*-glycans of bovine submaxillary mucin (BSM).

Peak No ^a^	Proposed Structure ^b^	Relative Quantity (%) ^c^ (Category) ^d^	Mass (*m/z*) ^e^		Mass Error (ppm) ^f^	Peak No ^a^	Proposed Structure ^b^	Relative Quantity (%) ^c^ (Category) ^d^	Mass (*m/z*) ^e^		Mass Error (ppm) ^f^
			Theoretical [M + H]^+^	Observed [M + H]^+^					Theoretical [M + H]^+^	Observed [M + H]^+^	
1	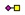	9.3 (Ma, 2, S)	633.2614	633.2611	0.4	18	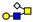	0.7 (Mi, 4, S, G, C3)	1014.3885	1014.3885	0.0
2	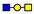	1.1 (Ma, 3, N, C1)	707.2982	707.2978	0.5	19	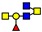	0.5 (Mi, 6, N, F, C4)	1259.5148	1259.5148	0.0
3	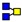	0.7 (Mi, 3, N, C4)	748.3247	748.3245	0.3	20	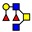	0.1 (Mi, 6, N, F, C4)	1202.4933	1202.4937	−0.3
4	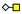	11.0 (Ma, 2, S)	649.2563	649.2560	0.4	21	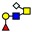	0.6(Mi, 5, S, F, C3)	1160.4464	1160.4464	0.0
5	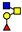	4.6 (Ma, 4, N, F, C2)	853.3561	853.3558	0.3	22	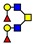	0.4 (Mi, 7, N, F, C4)	1364.5462	1364.5465	−0.2
6	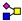	32.6 (Ma, 3, S, C3)	836.3408	836.3399	1.0	23	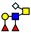	0.1 (Mi, 6, S, F, C3)	1306.5043	1306.5033	0.8
7	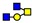	1.7 (Ma, 4, N, C2)	910.3775	910.3770	0.6	24	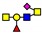	0.3 (Mi, 6, S, F, C3)	1347.5309	1347.5308	0.1
8	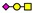	1.5 (Ma, 3, S, C1)	795.3142	795.3137	0.6	25	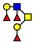	0.1 (Mi, 7, N, F, C2)	1307.5247	1307.5247	0.0
9	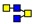	0.4 (Mi, 4, N, C4)	951.4041	951.4039	0.2	26	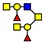	0.1 (Mi, 8, N, F, C2)	1567.6255	1567.6254	0.1
10	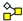	20.5 (Ma, 3, S, C5)	852.3357	852.3349	1.0	27	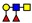	- (T, 5, N, F, C3)	1098.5188	1098.5190	−0.2
11	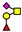	1.0 (Ma, 4, S, F, C1)	941.3721	941.3721	0.0	28	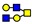	- (T, 5, N, G, C2)	1171.5352	1171.5353	−0.1
12	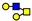	0.7 (Mi, 4, N, G, C2)	869.3510	869.3505	0.6	29	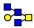	- (T, 5, N, G, C4)	1212.5617	1212.5617	0.0
13	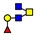	3.1 (Ma, 5, N, F, C4)	1056.4354	1056.4353	0.1	30	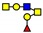	- (T, 6, N, F, C2)	1317.5931	1317.5933	−0.2
14	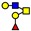	2.2 (Ma, 5, N, F, G, C2)	1015.4089	1015.4089	0.0	31	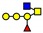	- (T, 6, N, F, G, C2)	1276.5665	1276.5665	0.0
15	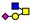	2.3 (Ma, 4, S, C2)	998.3936	998.3931	0.5	32	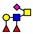	- (T, 6, S, F, C3)	1389.6142	1389.6145	0.0
16	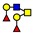	2.4 (Ma, 6, N, F, C2)	1161.4668	1161.4667	0.1	33	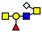	- (T, 6, S, F, C3)	1462.6306	1462.6305	0.0
17	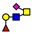	2.0 (Ma, 5, S, F, C3)	1144.4515	1144.4514	0.1	34	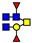	- (T, 7, N, F, C2)	1504.6775	1504.6773	0.2

^a^ Peak numbers correspond to peak numbers in Figure 1. ^b^ ■, GlcNAc; ■, GalNAc; ●, Gal; ▲, Fuc; ◆, Neu5Ac; ◇, Neu5Gc. ^c^ Quantity (%) of each AB-labeled major and minor *O*-glycan (Peaks 1–26) relative to total *O*-glycans was determined from the EIC areas for all observed charge states and the total EIC area for all *O*-glycans (as 100%) using LC–ESI–HCD–MS/MS. Trace *O*-glycans (Peaks 27–34) were obtained from ProA-labeled *O*-glycans. ^d^ Major (Ma, >1.0%; relative quantities of each glycan among total *O*-glycans as 100%), minor (Mi, 0.1%–1.0%), and trace (T, <0.1%) *O*-glycans; di- (2), tri- (3), tetra- (4), penta- (5), hexa- (6), hepta- (7), and octa- (8) saccharides; sialylated (S), neutral (N), fucosylated (F), and terminal-galatosylated (G) *O*-glycans; core 1 (C1), core 2 (C2), core 3 (C3), core 4 (C4), and core 5 (C5) structure types. ^e^ All *O*-glycan mass values were calculated and observed as singly charged forms. ^f^ Mass error was calculated as [(observed mass − theoretical mass)/theoretical mass] × 10^6^ (a high mass accuracy of glycan; <10.0 ppm).

**Table 2:** Summary of *O*-glycopeptides, *O*-glycosylation sites, and attached *O*-glycans identified from proteinase K-digested BSM.

Name	*O*-Glycopeptide ^a^	Peptide Mass (*m*/*z*)		*O*-Glycopeptide Mass (*m*/*z*) ^b^			Attached *O*-Glycan ^d^	Glycan No ^e^
		Theoretical [M + H]^+^	Observed [M + H]^+^	Theoretical [M + H]^+^	Observed [M + H]^+^	Error (ppm)^c^		
GP1	_307_RPSYGAL	763.4097	763.4146	730.8356	730.8362	0.8	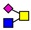	G6
GP2	_625_QTLGPL	628.3665	628.3776	663.3140	663.3190	7.7	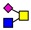	G6
GP3	_1080_RPEDNTAVA	972.4745	972.4632	835.3680	835.3622	−6.9	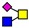	G6
GP4	_307_RPSYGAL	763.4097	763.4107	738.8330	738.8342	1.5	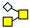	G10
GP5	_625_QTLGPL	628.3665	628.3776	671.3114	671.3165	7.6	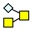	G10
GP6	_1080_RPEDNTAVA	972.4745	972.4631	843.3654	843.3608	−5.5	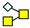	G10
GP7	_625_QTLGPL	628.3665	628.3773	561.7743	561.7794	9.1	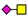	G1
GP8	_625_QTLGPL	628.3665	628.3783	825.3668	825.3721	6.5	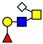	G21
GP9	_1080_RPEDNTAVA	972.4745	972.4621	1046.9550	1046.9504	−4.4	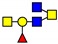	G19
GP10	_56_SGETRTSVI	949.4949	949.4953	722.3385	722.3384	−0.1	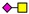	G1
GP11	_272_GSPSSVSSAEQI	1148.5430	1148.5795	923.4022	923.4198	19.0	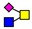	G6
GP12	_728_TMTTRTSVVV	1094.5874	1094.5842	1152.0195	1152.0195	0.0	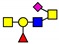	G24
GP13	_259_SHSSSGRSRTI	1174.5923	1174.6086	1017.4533	1017.4581	4.7	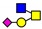	G15
GP14	_56_SGETRTSVI	949.4949	949.4957	730.3359	730.3360	0.1	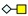	G4
GP15	_272_GSPSSVSSAEQI	1148.5430	1148.5803	931.3997	931.4170	18.6	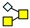	G10
GP16	_728_TMTTRTSVVV	1094.5874	1094.5839	1160.0169	1160.0167	−0.2	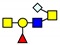	G33
GP17	_259_SHSSSGRSRTI	1174.5923	1174.6099	1025.4508	1025.4579	7.0	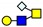	G18

^a^*O*-glycosylation sites are presented with bold underlined **S** or **T**. ^b^ All glycopeptide masses were calculated and observed as doubly charged forms. ^c^ Mass error was calculated as [(observed mass − theoretical mass)/theoretical mass] × 10^6^ (a high mass accuracy of glycopeptide; <20.0 ppm). ^d^ ■, GlcNAc; ■, GalNAc; ●, Gal; ▲, Fuc; ◆, Neu5Ac; ◇, Neu5Gc. ^e^ Glycan numbers correspond to peak numbers in Figure 1 and Table 1.

## Supplementary Materials

The following are available online at https://www.mdpi.com/2218-273X/10/4/636/s1, Figure S1: Precursor ion of G24 (theoretical *m*/*z* = 1347.5309) in the HCD-MS spectrum (full mass scan mode); Figure S2: Precursor ions of (**A**) GP8 (theoretical *m*/*z* = 825.3721), (**B**) GP9 (theoretical *m*/*z* = 1046.9504), (**C**) GP12 (theoretical *m*/*z* = 1152.0195), and (**D**) GP16 (theoretical *m*/*z* = 1160.0167) in the HCD–MS spectrum (full mass scan mode).

## Abbreviations

AA	amino acid
AB	2-aminobenzamide
BSM	bovine submaxillary mucin
EIC	extracted ion chromatogram
ESI	electrospray ionization
Fuc	fucose
Gal	galactose
GalNAc	*N*-acetylgalactosamine
GlcNAc	*N*-acetylglucosamine
HCD	high-energy collisional dissociation
Hex	hexose
HexNAc	hexosamine
LC	liquid chromatography
MALDI	matrix-assisted laser desorption/ionization
MS	mass spectrometry
MS/MS	tandem mass spectrometry
Neu5Ac	*N*-acetylneuraminic acid
Neu5Gc	*N*-glycolylneuraminic acid
*O*-glycan	Ser/Thr-linked glycan
PMP	1-phenyl-3-methyl-5-pyrazolone
ProA	procainamide
SA	sialic acid
SDS-PAGE	sodium dodecyl sulfate-polyacrylamide gel electrophoresis
TIC	total ion chromatogram
TOF	time-of-flight
UPLC	ultra-performance liquid chromatography
