# Supplementary material for: Structural and Quantitative Characterization of Mucin-Type O-Glycans and the Identification of O-Glycosylation Sites in Bovine Submaxillary Mucin
Source: Biomolecules. 2020 Apr 20;10(4):636. doi: 10.3390/biom10040636 (PMC7226346; doi:10.3390/biom10040636)
Supplement: Supplementary file 1 [file biomolecules-10-00636-s001.pdf]

biomolecules

Article-Supplementary Materials

Structural and Quantitative Characterization of Mucin-type *O*-glycans and the Identification of *O*-glycosylation Sites in Bovine Submaxillary Mucin

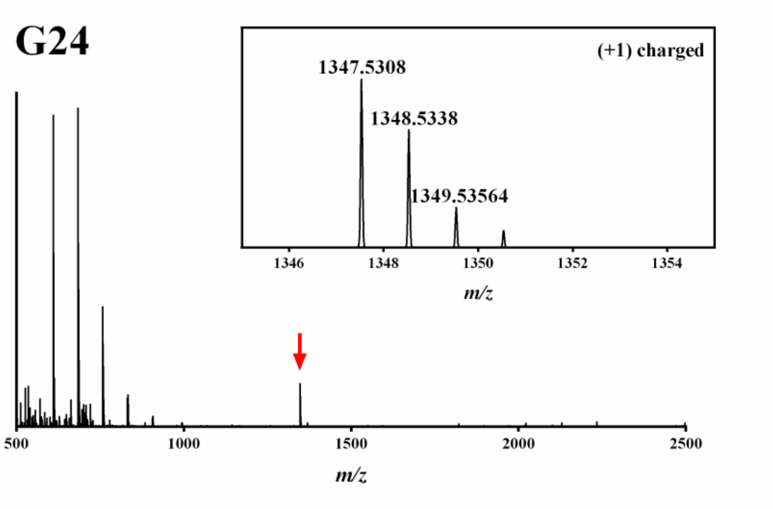

Figure S1. Precursor ion of G24 (theoretical m/z = 1347.5309) in the HCD-MS spectrum (full mass scan mode).

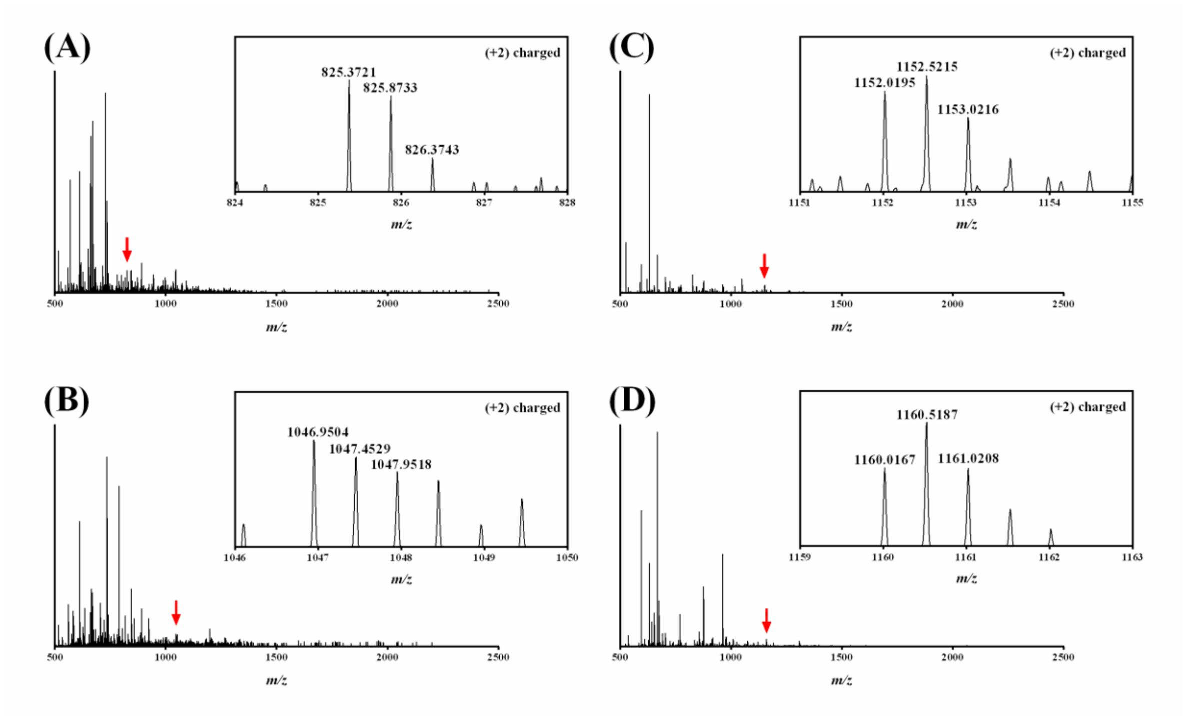

Figure S2. Precursor ions of (A) GP8 (theoretical *m/z* = 825.3721), (B) GP9 (theoretical *m/z* = 1046.9504), (C) GP12 (theoretical *m/z* = 1152.0195), and (D) GP16 (theoretical *m/z* = 1160.0167) in the HCD-MS spectrum (full mass scan mode).
